# Identification of gait domains and key gait variables following hip fracture

**DOI:** 10.1186/s12877-015-0147-4

**Published:** 2015-11-18

**Authors:** Pernille Thingstad, Thorlene Egerton, Espen F. Ihlen, Kristin Taraldsen, Rolf Moe-Nilssen, Jorunn L. Helbostad

**Affiliations:** Department of Neuroscience, Norwegian University of Science and Technology, PO Box 8905, 7491 Trondheim, Norway; Department of Global Public Health and Primary Care, University of Bergen, Bergen, Norway; Department of Clinical Services, St. Olav University Hospital, Trondheim, Norway

**Keywords:** Gait, Hip fracture, Factor analysis, Rehabilitation

## Abstract

**Background:**

Restoration of gait is an important goal of rehabilitation after hip fracture. Numerous spatial and temporal gait variables have been reported in the literature, but beyond gait speed, there is little agreement on which gait variables should be reported and which are redundant in describing gait recovery following hip fracture. The aims of this study were to identify distinct domains of gait and key variables representing these domains, and to explore how known predictors of poor outcome after hip fracture were associated with these key variables.

**Methods:**

Spatial and temporal gait variables were collected four months following hip fracture in 249 participants using an electronic walkway (GAITRite®). From the initial set of 31 gait variables, 16 were selected following a systematic procedure. An explorative factor analysis with oblique (oblimin) rotation was performed, using principal component analysis for extraction of factors. Unique domains of gait and the variable best representing these domains were identified. Multiple regression analyses including six predictors; age, gender, fracture type, pain, global cognitive function and grip strength were performed for each of the identified key gait variables.

**Results:**

Mean age of participants was 82.6 (SD = 6.0) years, 75 % were women, and mean gait speed was 0.6 (SD = 0.2) m/sec. The factor analysis revealed four distinct gait domains, and the key variables that best represented these domains were double support time, walk ratio, variability of step velocity, and single support asymmetry. Cognitive decline, low grip strength, extra capsular fracture and male gender, but not pain or age, were significant predictors of impaired gait.

**Conclusions:**

This work proposes four key variables to represent gait of older people after hip fracture. These core variables were associated with known predictors of poor outcome after hip fracture and should warrant further assessment to confirm their importance as outcome variables in addition to gait speed.

**Electronic supplementary material:**

The online version of this article (doi:10.1186/s12877-015-0147-4) contains supplementary material, which is available to authorized users.

## Background

Safe and efficient gait is a prerequisite for independent living in old age. Worldwide there are 1.6 million hip fractures annually [1]. The majority of hip fracture patients never regain prefracture function [2]. Gait impairment is an important reason this group faces long-term disability [3], loss of independence in activities of daily living (ADL), and increased fall risk [4].

The underlying mechanisms for gait decline following hip fractures are poorly understood and there are few reports on gait characteristics beyond gait speed in this group. Gait speed has been recommended as an overall measure of health and function in older adults [5]. However gait is not a unitary concept, and different gait variables have demonstrated discriminate and predictive ability for cognitive function [6] and for falls [7] suggesting there are complementary information to gain from gait variables beyond gait speed.

With the advent of electronic walkways, a large number of gait variables can be easily measured and reported, even in frail populations such as hip fracture patients. Identification of which gait variables capture the most important properties of gait impairment after hip fracture would aid future research targeting gait.

Factor analysis can be used to explain the underlying structure of a set of variables and thereby reduce a large dataset to a more manageable size, while retaining as much of the original information as possible [8]. Previous studies deriving gait domains by use of factor analysis in relatively healthy community-dwelling older adults have demonstrated three to six distinct domains of gait [6, 9, 10]. However, it is not known if the same domains are representative for gait in frailer older people following hip fracture. The present study aimed to identify a set of gait variables to describe gait in hip fracture patients and to explore how known risk factors for poor outcome after hip fracture are associated with these gait variables.

## Methods

### Participants

Data were collected between April 2008 and December 2011 from participants included in the Trondheim Hip Fracture Trial [11]. Inclusion criteria for that trial were community-dwelling prior to the fracture, aged ≥ 70 years, and having undergone surgery for intra- or extra-capsular hip fracture (ICD 72.0–72.2). Exclusion criteria were pathological fractures and life expectancy shorter than 3 months. For the present study, data from the assessment carried out four months post-surgery were used.

The Trondheim Hip Fracture Trial was approved by the Regional Committee of Ethics in Medical Research (REK4.2008.335), the Norwegian Social Science Data Services (NSD19109), and the Norwegian Directorate of Health (08/5814). Patients or their next-of-kin gave informed written consent to be included in the study before participation.

### Procedure

Gait assessment was carried out using a GAITRite® mat (CIR systems Inc. Sparta, US). Data were collected from a 4.88 m active area in the middle of an 8.0 m walkway. Participants walked back and forth at self-selected preferred speed, with each walk starting from a standing position approximately 1.5 m outside the active area. Walking aids were permitted only when the participant was unable to walk without one. Where two walks were available, the values from each walk were averaged.

### Outcomes

Basic ADL was assessed using the Barthel Index [12] and instrumental ADL function using Nottingham Extended I-ADL scale [13]. Global cognitive function was assessed using the Mini Mental State Examination (MMSE) [14]. Grip strength was measured by the Jamar® handheld dynamometer, using the maximum value of two attempts by the strongest hand. Level of pain in the affected hip while walking was measured using an eleven-point numeric rating scale. Fracture type was dichotomised into intra- and extra-capsular fractures.

#### Data processing and statistical analysis

Data from the GAITRite mat were processed using the PKmas® software, which is a new programme developed to improve the processing of difficult footstep patterns, such as overlapping steps. Outcomes derived from the PKmas and GAITRite softwares have been shown to be comparable at group level for most variables [15]. Mean, within subject standard deviation (SD), coefficient of variance (CV (SD/mean*100)), and left/right ratio of spatial and temporal gait variables were calculated by the software and exported to Microsoft Excel® for further calculations of walk ratio (step length/cadence) and asymmetry: 100x(|ln(left/right)|) [16]. For the variability measures, the standard deviations for left and right sides were calculated separately and then averaged to avoid the effect of asymmetry on the values.

### Selection of gait variables

Thirty-one commonly reported gait variables were initially considered for the factor analysis. These included three broad categories of variables; the mean temporal and spatial values measured over multiple steps, variability over these steps measured as both SD and CV, and left-right step asymmetry. CV is preferred when increase in SD is proportional to the within subject mean value. If SD is unrelated to the within subject mean value SD should be used as the measure of variability [17]. Steps instead of strides allow for calculation of left/right asymmetry and were therefore chosen.

The pattern of correlation among variables within a dataset determines if factor analysis is a suitable method. The correlation matrix determinant was checked for indications of multicollinearity (should be >0.00001) and the Kaiser-Meyer-Olkin statistics (KMO) for sampling adequacy (should be >0.5 for individual variables and >0.7 for overall KMO) [8]. Variables with a correlation higher than 0.9 and with KMO below 0.5 were considered removed from the analysis.

Each gait variable was inspected for normal distribution by Q-Q plots. As factor analysis is not very sensitive to deviations from the normal distribution [8], minor deviations were accepted. Based on the Q-Q plots, double support time, step time and all the variability variables except SD step width, were log transformed.

### Factor analysis

An exploratory factor analysis was conducted in SPSS (IBM statistics 21). The extraction method was principal component analysis and the number of factors based on Eigenvalues > 1. Factors were expected to be correlated, and therefore oblique rotation used [8]. Criteria for selection of key variables were high factor loading in combination with low levels of cross loading. Factor loadings higher than 0.3 was set as the limit for cross loadings [8].

In order to assess the robustness of the results, we performed additional analyses using gait data collected twelve months following the fracture and also after exclusion of participants who walked with walking aids during the assessment. We also performed an additional factor analysis using a similar set of variables as Lord et al. [9], in order to compare with findings in healthy older adults.

### Multiple regression analyses

Multiple regression analyses were carried out with gait speed and each of the identified key gait variables as dependent variables. Six known risk factors for poor outcome after hip fracture (age, gender, fracture type, pain level, grip strength and MMSE score) were entered as predictors. We used log transformed values for skewed variables (double support time and step velocity variability).

## Results

Two hundred and forty nine participants were included in the analysis. Seventy-five percent were women. Time since fracture was 16.2 (SD 1.8 weeks). Sixty-four percent had intracapsular fractures, and of these the majority (67 %) were operated with arthroplasty. Sixty percent of the participants used walking aids indoors. Twenty-five percent were not able to walk without walking aids during the gait assessment and therefore used either a rollator or a stick. Table 1 shows clinical characteristics of the participants four months post-surgery, while Table 2 shows means, standard deviations and the range for the gait variables included in the factor analysis.

**Table 1:** Clinical characteristics four months post-surgery

Sample characteristics	Number	Mean (SD)
Age (years)	249	82.6 (6.0)
Barthel Index (0–20)	249	17.4 (3.0)
Nottingham E-ADL Index (0–66)	248	35.9 (17.0)
Mini Mental State Examination (0–30)	247	24.3 (5.2)
Grip strength women	185	18 (5)
Grip strength men	61	30 (8)
Pain (0–10)	240	1.8 (2.0)
	n/N	%
Women	191/249	77
Intracapsular fractures	158/249	64
Arthroplasty^a^	107/158	68

*E-ADL* Extended activities of daily living, ^a^proportion of intracapsular fractures

**Table 2:** Gait characteristics four months post-surgery (*n* = 249)

Mean gait characteristics	Mean	SD	Range
Steps (number)	25.2	10.2	8–83
Speed (m/sec)	0.62	0.25	0.20–1.42
Cadence (steps/min)	91	16	55–132
Walk Ratio (step length/cadence)	0.45	0.13	0.11–0.81
Step length (m)	0.40	0.12	0.13–0.81
Step width (cm)	8.93	3.9	0.35–22.0
Step time (s)	0.682	0.128	0.455–1.099
Single support (%)	30.8	4.6	17.4–38.9
Double support (s)	0.536	0.197	0.210–1.159
Variability gait characteristics	Mean	SD	range
SD step velocity (m/sec)	0.05	0.02	0.02–0.11
SD step length (m)	0.03	0.01	0.01–0.08
SD step width (cm)	1.9	0.7	0.6–4.1
SD step time (s)	0.047	0.039	0.007–0.319
SD single support (s)	0.036	0.018	0.007–0.126
SD double support (s)	0.060	0.067	0.008–0.627
Asymmetry gait characteristics %	Mean	SD	range
Step length asymmetry	15	21	0–163
Step time asymmetry	10	10	0–46
Single support time asymmetry	14	15	0–76

*SD* standard deviation, *CV* coefficient of variation (SD/mean x 100); Asymmetry = 100 x (|ln(left/right)|)

### Initial selection of variables

**Fig. 1:**
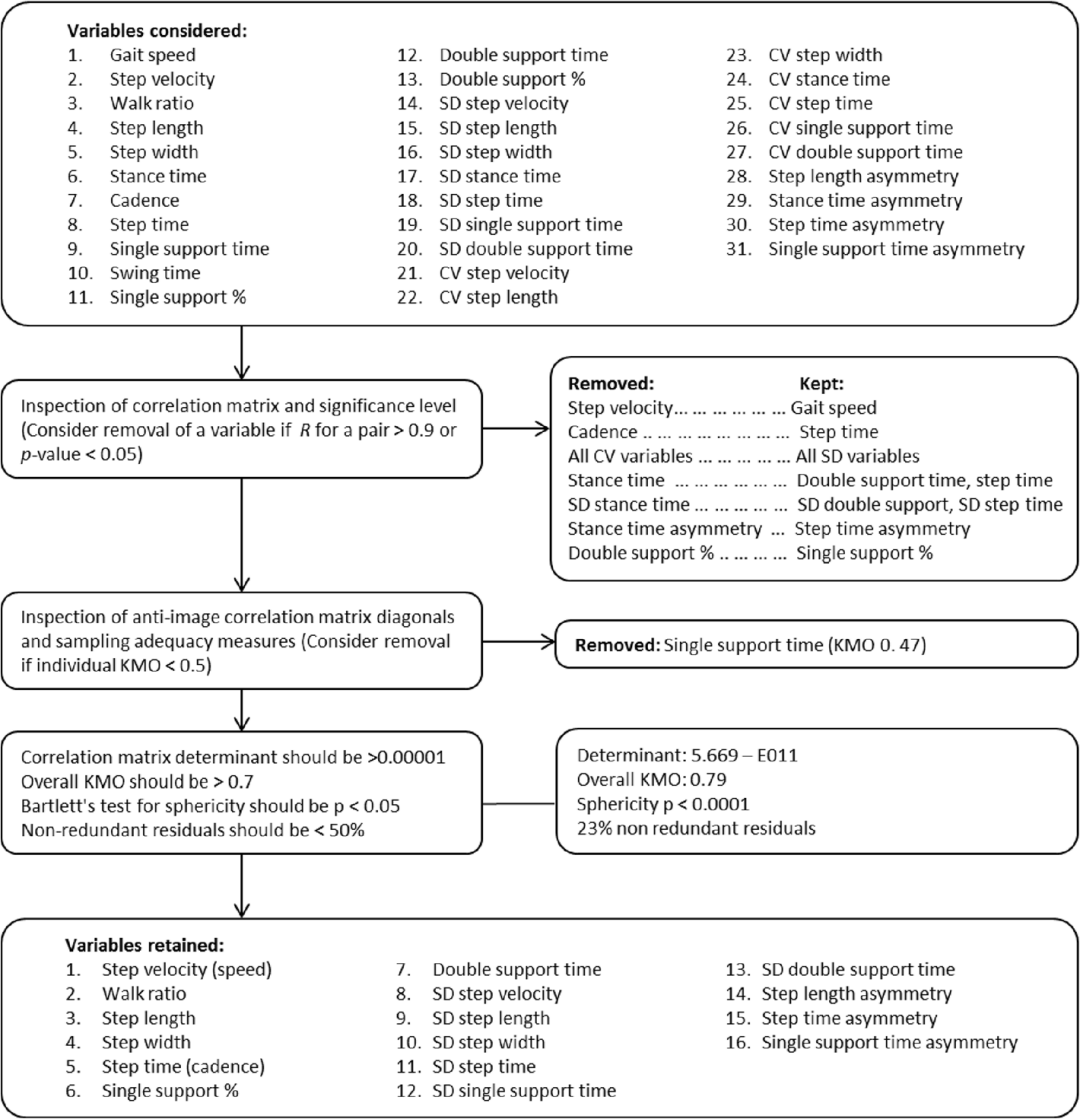
Flow chart describing the process for initial selection of variables for the factor analysis

The procedure for selection of variables is presented in Fig. 1. Based on inspection of the correlation matrix and KMO statistics for individual variables, seven variables were removed including cadence, all stance time parameters, percentage double support, and single support time. Variability was reported as SD based on inspection of degree of proportionality between SD and means, and CV not included.

The selection procedure resulted in 16 variables remaining to be included in the factor analysis (Table 2). For this model the overall KMO was 0.79 and the Bartlett’s test of sphericity was significant (p < 0.0001).

### Factor analysis

The factor analysis (Table 3) yielded four domains explaining 79 % of the variance. These domains were labelled in line with earlier published models [9]. Domain 1: Pace/rhythm, Domain 2: Postural control, Domain 3: Variability, and Domain 4: Asymmetry. Forty-seven percent of the variance was explained by the pace/rhythm domain which also contained the highest number of variables and was dominated by mean and variability of temporal variables. Postural control explained 15 %, variability 11 % and asymmetry 7 %. The pace/rhythm, postural control and asymmetry domains had about 10 % overlap in variance between factors, thus supporting the use of oblique rotation. Cross loadings for single variables above 0.3 were found for step velocity, step time, single support percentage, step length and SD step length.

**Table 3:** Factor loadings and proportion of variance explained by each domain for the 16 gait variables included in the analysis

	Pace/Rhythm 47 %	Postural control 15 %	Variability 11 %	Asymmetry 7 %
Pace/Rhythm:				
Gait speed	**-.721**	**-.353**	.218	-.110
Step time	**.978**	**-.394**	-.224	-.052
Single support %	**-.495**	**-.473**	.161	-.224
Double support time	**.857**	.108	-.194	.100
SD step time	**.888**	.057	.162	.066
SD single support time	**.847**	.031	.168	.028
SD double support time	**.855**	.108	.129	.048
Postural control:				
Walk ratio	-.135	**-.900**	.019	-.177
Step length	-.436	**-.653**	.155	-.148
Step width	.051	**.635**	.122	-.078
Variability:				
SD step velocity	.036	.237	**.820**	.095
SD step length	**.508**	.149	**.660**	.028
SD step width	-.159	-.163	**.666**	-.013
Asymmetry:				
Step length asymmetry	-.062	.215	-.070	**.725**
Step time asymmetry	.028	-.121	.089	**.935**
Single support asymmetry	.024	-.109	.016	**.953**

Factor loadings above 0.3 are in bold

*SD* standard deviation

Four variables with high loadings without cross loading were found for the pace/rhythm domain; double support time and SD of single support time, double support time and step time. For the other three domains walk ratio, variability in step velocity and asymmetry of single support time were the variable with highest loading and with no cross loadings.

Double support time were selected above measures of variability to represent the Pace/Rhythm domain due to previous work indicating the clinical relevance of this variable [7, 18–21] and as mean of temporal gait variables more consistently has demonstrated good reliability as compared to measures of gait variability [22].

Additional analyses firstly excluding participants using walking aids during the assessments and secondly using the data from the 12-month assessments, revealed the same domains and similar loadings as for the primary analysis (Additional file 1). Using the variables similar to Lord et al. [9], the factor analysis revealed almost the same factor structure as found in healthy older adults, except that with our hip fracture patients’ data the pace and rhythm domains were combined (Additional file 2).

### Multiple regression analyses

Results from the multiple regression analyses are presented in Table 4 showing that male gender and extra capsular fractures were associated with lower gait speed, increased double support time and higher asymmetry. Reduced global cognitive function were associated with lower gait speed and increased double support time, and low grip strength with reduced gait speed, increased double support time and lower walk ratio. Age and pain were not significantly associated either of the key variables.

**Table 4:** Multiple linear regression analysis with the key gait variables and gait speed as dependent variable

Four months Adjusted R^2^	Double support 0.150		Walk ratio 0.182		SD step velocity .008		SS asymmetry .074		Speed .240	
	B	p	B	p	B	p	B	p	B	p
age	.087	.186	-.113	.080	.114	.107	.048	.481	.113	.070
gender	-.227	*.007*	-.087	.288	.031	.735	-.200	*.022*	.201	*.011*
fracture	.160	*.008*	.008	.892	-.063	.330	.199	*.002*	-.190	*.001*
pain	.026	.672	-.076	.199	-.024	.718	.115	.070	-.060	.296
MMSE	-.208	*.001*	.100	.104	-.125	.067	.014	.827	.205	*.001*
grip strength	-.267	*.003*	.296	*.001*	.100	.309	-.094	.320	.374	*<.001*

*MMSE* mini mental state examination, *SS* single support

## Discussion

This study aimed to find the unique domains that characterise gait in hip fracture patients and the key gait variables that best represent each of these domains. As expected we found high correlations among gait variables captured from the same walk. Despite this, the factor analysis revealed four relatively distinct domains and at least one variable for each domain with high factor loading and minimal cross loadings. The relevance of the four key gait variables was supported by the regression analysis, showing associations with established predictors for poor outcome following a hip fracture.

The main structure of the factor solution found in the present work was similar to that found in a previous sample of community-dwelling older people, supporting the notion of a more universal gait model [23]. As a result we choose to name domains revealed from the factor analysis in our study according to the previous models; pace/rhythm, postural control, variability and asymmetry.

In line with earlier work we found that temporal variables, mean step width, asymmetry in temporal variables, and spatial variability loaded to unique gait domains. However we did not find pace (velocity and step length) and rhythm (temporal variables) separated onto unique domains. Cross loadings were found for step velocity, step length and percentage single support. These are variables highly related to gait speed suggesting that these variables represent overall gait performance similarly to gait speed.

We found temporal and spatial variability loaded onto separate domains. This was the same finding as in healthy older adults [9]. Previous work has also demonstrated low correlation between these gait characteristics and suggested that variability in temporal and spatial gait characteristics represent different constructs [17].

We also included walk ratio which is step length/cadence, as it has earlier demonstrated to be relatively independent of gait speed, which step length and cadence are not [24]. Walk ratio loaded strongly to the postural control domain, and was also found to be associated with grip strength, which is often seen as a general indicator of health. Furthermore, temporal and spatial asymmetry between the affected and non-affected leg was also included as asymmetry is of particularly importance when assessing recovery of gait after a uni-lateral injury. Asymmetry loaded to a separate domain and was also found to be related to fracture type, thus confirming the importance of assessing this variable following hip fracture. Despite being derived from other variables walk ratio and asymmetry demonstrated to represent different constructs and be relatively independent of the original variables and could be of clinical importance in this population.

As in most previous studies, the regression analysis demonstrated that gait speed might be a robust indicator of gait. However, gait speed did not have a high loading in the variability and the asymmetry domains, suggesting that these domains represented by the key variables step velocity variability and single support asymmetry have added value beyond gait speed.

The clinical correlates of the four domains cannot be implied from the factor analysis, but has to be interpreted in view of empirical evidence and earlier findings. Pace and rhythm in gait have been suggested to reflect central gait control mechanisms, with ‘pace’ being related to higher cortical mechanisms and ‘rhythm’ to spinal and brain stem mechanisms [6]. The ratio of step length to cadence (walk ratio) in normal gait is highly consistent across speeds and has been suggested to reflect higher order automatic control of gait [25]. Low walk ratio has been associated with cautious gait [24] and falls [26]. A combination of shorter step length, increased cadence and broader step width, rather than simply reducing gait speed, has been described as a strategy to cope with medio-lateral balance perturbations and increased medio-lateral margins of stability during walking [27, 28]. Hip fracture is a unilateral injury associated with pain [29], changes in biomechanics and muscle function of the hip abductors [30]. Asymmetric weight loading is a persistent characteristic of gait after hip fracture [31], and high levels of gait asymmetry were also found in this study.

The regression analyses indicated an association between gait impairments and known predictors of poor functional outcome after hip fracture including cognitive function, male gender, fracture type, and grip strength which is associated with sarcopenia and frailty [32]. This suggests that the identified key gait variables are relevant to outcome following hip fracture and can thus be recommended for the assessment of gait following a hip fracture. Further work should explore more specific hypotheses including how cognitive functioning, physical frailty and muscle function related to hip stability are associated with the different key gait variables and look specifically at how each of the key gait variables respond to interventions.

The study has some possible limitations. A factor solution is the result of the selection of variables entered into the analysis. Therefore, it is possible that other gait variables than those included in our model are important for outcome after hip fracture. Further this work included a heterogenic sample with regards to physical and cognitive function. This should make results generalizable but could also hide differences between subgroups. Never-the-less our gait model was found to be robust as demonstrated by similar findings with the 12-months post-fracture data and if participants walking with walking aids were excluded. Furthermore, the structure of the factor solution and loading of variables is also very similar to the model previously described in healthy older adults [9].

## Conclusion

The present work suggests four key gait variables: double support time, walk ratio, SD of step velocity and single support time asymmetry to represent the unique domains of gait in older hip fracture patients. It is suggested that the findings may facilitate the selection and interpretation of gait variables in future clinical trials.

Further work is needed to determine how these variables are associated with clinical features, or can be used to provide insight into the improvement in gait performance achieved by different interventions. In the longer term in-depth knowledge about gait characteristics could help to guide the development of more targeted and effective interventions to maximise gait recovery and to understand underlying mechanisms of gait impairments in older hip fracture patients.

### Data availability

Data files are not available due to participants’ confidentiality.

## Additional files

Additional file 1:
**Factor solution based on the 12 months dataset.** (DOCX 38 kb) Additional file 2:
**Factor solution based on replication of the Sue Lord model.** (DOCX 39 kb)

## Abbreviations

ADL: Activities of Daily Living
MMSE: Mini Mental State Examination
CV: Coefficient of variance
Walk Ratio: Step length (cm)/cadence (steps/min)
KMO: Kaiser-Meyer-Olkin statistics
