# Supplementary material for: Identification of gait domains and key gait variables following hip fracture
Source: BMC Geriatr. 2015 Nov 18;15:150. doi: 10.1186/s12877-015-0147-4 (PMC4652377; doi:10.1186/s12877-015-0147-4)
Supplement: Additional file 1: — Factor solution based on the 12 months dataset. (DOCX 38 kb) [file 12877_2015_147_MOESM1_ESM.docx]

Additional file 1. The pattern matrix of the oblimin rotated solution showing factor loadings and proportion of variance explained by each domain. Factor loadings above 0.3 in bold. Dataset: 12 months assessment

|  | **Pace/ rhythm** | **Postural control** | **Variability** | **Asymmetry** |
| --- | --- | --- | --- | --- |
| Step velocity | **-.799** | **.331** | .049 | .009 |
| Step time (cadence) | **.932** | **.401** | -.152 | .129 |
| Single support %  Pace /Rhythm  47% | **-.688** | **.348** | .092 | -.075 |
| Double support time | **.855** | .049 | -.154 | .142 |
| SD step time | **.819** | -.066 | .281 | .085 |
| SD single support time | **.685** | -.081 | **.308** | .138 |
| SD double support time | **.737** | .081 | .123 | .135 |
|  |  |  |  |  |
| Walk Ratio  Postural control  10% | -.083 | **.885** | -.033 | .004 |
| Step length | **-.605** | **.593** | .025 | .001 |
| Step width | -.135 | **-.644** | .051 | .167 |
|  |  |  |  | CONTROL  47%  ADAPTIVE STRATEGIES 15%  VARIABILITY  11%  ASYMMETRY  7% |
| SD step velocity  Variability  12% | .045 | -.202 | **.851** | -.083 |
| SD step length | **.410** | -.125 | **.737** | -.092 |
| SD step width | -.243 | .284 | **.609** | .123 |
|  |  |  |  |  |
| Step length asymmetry  Asymmetry  7% | .139 | **-.379** | -.054 | **.486** |
| Step time asymmetry | .080 | .005 | -.028 | **.901** |
| Single support time asymmetry | .055 | -.032 | .006 | **.896** |
