# Supplementary material for: Identification of gait domains and key gait variables following hip fracture
Source: BMC Geriatr. 2015 Nov 18;15:150. doi: 10.1186/s12877-015-0147-4 (PMC4652377; doi:10.1186/s12877-015-0147-4)
Supplement: Additional file 2: — Factor solution based on replication of the Sue Lord model. (DOCX 39 kb) [file 12877_2015_147_MOESM2_ESM.docx]

Additional file 2. Replication of the Sue Lord Model. The rotated component matrix of the varimax rotated solution showing factor loadings and proportion of variance explained by each domain. Factor loadings above 0.3 in bold. Dataset: 4 months assessment

|  | **Pace/ rhythm** | **Postural control** | **Variability** | **Asymmetry** |
| --- | --- | --- | --- | --- |
| Step velocity | **-.848** | **.367** | .187 | -.246 |
| Step time (cadence) | **.870** | **.339** | -.252 | .034 |
| Stance time | **.917** | .123 | -.222 | .127 |
| Single support time  Pace /Rhythm  35% | **.326** | **.832** | -.167 | -.229 |
| Double support time | **.855** | .049 | -.154 | .142 |
| SD step time | **.870** | **.339** | -.252 | .034 |
| SD single support time | **.853** | -.082 | .175 | .155 |
| SD double support time | **.737** | .081 | .123 | .135 |
| SD stance | **.889** | -.141 | .137 | .173 |
| Postural control  14% |  |  |  |  |
| Step length | **-.637** | **.632** | .101 | -.277 |
| Step width | .183 | **-.631** | .129 | .022 |
|  |  |  |  | CONTROL  47%  ADAPTIVE STRATEGIES 15%  VARIABILITY  11%  ASYMMETRY  7% |
| SD step velocity  Variability  12% | .071 | **-.335** | **.824** | .115 |
| SD step length | **.514** | -.239 | **.661** | .124 |
| SD step width | -.282 | .149 | **.652** | .048 |
|  |  |  |  |  |
| Step length asymmetry  Asymmetry  20% | .147 | **-.437** | -.076 | **.626** |
| Step time asymmetry | .145 | -.150 | .072 | **.851** |
| Single support time asymmetry | .179 | -.110 | .037 | **.936** |
| Stance time asymmetry | .163 | .066 | .073 | **.915** |
